# Predictors of delayed Antenatal Care (ANC) visits in Nigeria: secondary analysis of 2013 Nigeria Demographic and Health Survey (NDHS)

**DOI:** 10.11604/pamj.2017.26.124.9861

**Published:** 2017-03-03

**Authors:** Alhaji Abubakar Aliyu, Tukur Dahiru

**Affiliations:** 1 Department of Community Medicine, Ahmadu Bello University, Zaria, Nigeria

**Keywords:** Antenatal care, delay, maternal health, predictors, Nigeria

## Abstract

**Introduction:**

Antenatal Care (ANC) is an important component of maternal health and covers a wide range of activities with huge potential benefits for positive pregnancy out comes. However, large proportions of women do initiate ANC early resulting in adverse consequences.

**Methods:**

The study utilized the nationally-representative sample of women of reproductive age interviewed during the 2013 Nigeria DHS. Analysis was restricted to 20, 467 women aged 15-49 years who had a live birth in the five-year period prior to the survey. Multinomial logistic regression was performed using Stata v13 to determine significant factors related to timing of initiation of ANC. Relative risk ratio (RRR) was used to assess the strength of association between independent and dependent variables.

**Results:**

Overall, 27%, 62% and 12% of women initiated ANC in the first, second and third trimesters respectively. In both the two model, the findings reveal that maternal education, level of media exposure, region and place of residence are the uniform predictors of initiation of ANC; having health insurance is a significant predictor of third trimester ANC initiation relative to first to first trimester only. Within the categories of household wealth, levels of participation in household decision-making and region some categories are significant predictors while others are not.

**Conclusion:**

Maternal education, level of media exposure, region and place of residence are the uniform and consistent predictors of delay in ANC initiation. This suggests that girl-child education, universal health coverage and universal health insurance could be the interventions required to improve service utilization and maternal health.

## Introduction

Maternal mortality, the death of a woman during pregnancy, childbirth or in the 42 days after delivery remains a major challenge to health systems globally [1]. All regions of the world have experienced declines in maternal deaths but fell short of achieving MDG 5 [2]. Of the ten countries accounting for 59% of maternal mortality, six are in SSA in addition to Nigeria are DRC, Ethiopia, Kenya, Tanzania, Kenya and Uganda; the remaining four are, in addition to India, Pakistan, Indonesia and Bangladesh [3]. With only 2% of the world population, Nigeria contributes a disproportionate 19% to the total global burden of 303, 000 annual maternal deaths [3]. The lifetime risk of maternal deaths in SSA is 1 in 36 compared to 1 in 4900 in developed nations; 1 in 150 for Oceania; 1 in 760 for Latin America and 1 in 250 in the Caribbean [3]. The fact is that pregnancy always carries risk of unexpected complications and 15% of pregnancies everywhere are life threatening [4] and the utilization of maternal health services is associated with improved pregnancy outcomes [5], including reduced maternal and perinatal mortality [6–8]. Antenatal care is an important component of maternal health and covers a range of activities such as nutrition education, tetanus vaccination, malaria prophylaxis, HIV testing and counselling and thus has the potential for the prevention of HIV from mother to child (PMTCT). The prevention and treatment of malaria among pregnant women can significantly improve maternal health and fetal outcomes [9]. Studies have shown that women who started antenatal care attendance early and attended the recommended number were more likely to be assisted during delivery by a skilled attendant compared to those who started ANC late and also attended only few visits [10–12]. It is known that ANC may not have the potential to predict and avert obstetric emergencies during pregnancy and child birth; however it exposes the women to health education on risk factors and encourages them to deliver with skilled attendants and or even in a health facility. Recent studies reported that women who knew about risk factors were more likely to utilize health facilities for delivery than those without knowledge [11, 13]. The benefits of antenatal care includes prevention and treatment of any complications, emergency preparedness, birth planning, satisfying unmet nutrition, social, emotional and physical needs of pregnant woman, provision of patient education plus successful care and nutrition of the newborn, identification of high risk pregnancy and encouragement of male partner involvement in ANC [14]. It is therefore, important that the pregnant women start ANC early in their pregnancy to fully benefit from these interventions.

With improved understanding of the need for women to prepare physically, mentally and even logistically for childbirth, antenatal care is recognized as a key maternal service in improving wide range of health outcomes for women and children [15]. Thus, the revised Focused Antenatal Care (FANC) model of World Health Organization (WHO) recommends at least 4 ANC visits for uncomplicated pregnancies with the first visit starting before 16 weeks of gestation [16]. However, utilization of antenatal care (ANC) services are often limited or delayed in developing countries due to several reasons that have been reported previously [10, 17–22]. Availability, affordability and easy access to health facilities where antenatal care is offered increase utilization of antenatal care. Cultural beliefs and practices about pregnancy will have influence on antenatal care use in that it might lead to mothers attending antenatal care late or not even attending at all [23, 24]. Other studies showed that women’s ANC attendance is mediated by previous experiences and the quality of care at earlier antenatal care visits [25]. In Nigeria, according to recent report, the proportion of pregnant women who attended the recommended four ANC visits increased from 45% in 2008 to 51% in 2013 [26]. Thus it is obvious that despite the efforts put in place by all the three tiers of Government including the Midwifery service scheme (MSS) to improve ANC attendance major barriers and challenges still remain for timely uptake of ANC.

While a handful of previous Nigerian studies on utilization of maternal health services are institutionally-based and restricted to specific region/zone and involving small sample size [19, 21, 27–29], this study seeks to use a national representative data from Nigeria Demographic and Health Survey (NDHS) to determine predictors of delayed ANC attendance among Nigerian women of reproductive age group. The findings will guide policy makers in the implementation of interventions that will improve and increase ANC utilization and contribute to the promotion of maternal health in Nigeria.

## Methods

### Data

Data for this study came from NDHS 2013 which was a nationally representative sample of women in reproductive age group (15-49 years). A national sample of 40,320 households from 904 primary sampling units (PSU) was selected. All women aged 15-49 who were usual members of the selected households or who spent the night before the survey in the selected households were eligible for individual interviews. As with previous Demographic and Health Surveys, 2013 NDHS was to provide reliable information among others on maternal and child health, childhood and adult mortality levels. The survey provides reliable estimates for key indicators at national levels as well as for urban and rural areas for the 36 states and the Federal capital territory (FCT). Nigeria is administratively divided into 36 states and the FCT. The states are regrouped into six geo-political zones (North West, North Central, North East, South West, South East and South South). Each state is subdivided into local government areas (LGAs), of which there are 774 and each LGA is further subdivided into smaller (secondary and tertiary) localities. During the 2006 census, each locality was subdivided into enumeration areas (EAs), however, the EAs in Nigeria are small in size with an average of 211 inhabitants (48 households), so the 2013 DHS included several EAs per DHS cluster (with a minimum cluster size of 80 households). The NDHS sample was stratified and selected independently in three stages from sampling frame. Each state was stratified into urban and rural areas.

In the first stage 893 localities were selected with probability proportionate to size and with independent selection in each sampling stratum. In the second stage, one EA was randomly selected from most of the selected localities with an equal probability selection. In a few larger localities, more than one EA was selected, giving a total of 904 EAs that were selected. Household listing operation of selected 904 EAs was done before the main survey, drawing a location map and a detailed sketch map and recording on the household forms all occupied residential households found in EA with address and the name of household head. Where a selected EA had less than 80 household, a neighboring EA from the selected locality was added to the cluster and listed completely. This list of households served as the sampling frame for the selection of households in the third stage. Finally, in the third stage a fixed number of 45 households were selected in every urban and rural cluster through equal probability systematic sampling based on the newly updated household listing. The sample allocation features an equal size allocation with minor adjustments. Among the 904 clusters, 372 were urban and 532 rural; each with a total number of households of 16, 740 and 23, 940 respectively. This gives overall total households sampled at 40, 680. Since 2013 NDHS used a 3 stage stratified cluster sampling technique, it means sampling weights based on sampling probability will be required for any data analysis to ensure the representativeness of the survey results at both national and domain levels. For a detailed description of the sampling procedures, distribution of population, EAs by state and the survey questionnaires see NDHS report (26).

### Variables

The primary outcome variable for this study is delayed ANC (defined as having the first ANC in second or third trimester). From 2013 Nigeria DHS, the following potential factors associated with delay in seeking ANC as predictor variables were identified: pregnancy intention; maternal age at birth of last child; maternal and spousal level of education; maternal and spousal occupational statuses; religion; birth order; household wealth level; level of participation in household decision making; exposure to source of health information (through the three media channels of radio, television and newspaper/magazine); type of marriage; ethnicity; geopolitical zone; place of residence and insurance cover.

### Statistical analysis

The first stage of the statistical analysis involved an examination of associations between the outcome variable and various socio-demographic factors; this involved conducting chi-squared tests. Further, a test of collinearity was conducted to determine if the variables in the model are correlated with one another and the variable strongly related with the outcome variable was retained. Collinearity was further tested with variance inflation factor (VIF) and any variable that overshoots the threshold was dropped from the model. Finally, polytomous logistic regression was carried out to determine significant factors related to timing of initiation of antenatal care. This model was selected since the outcome variable has more than two categories in contrast binary logistic regression where the outcome variable has two categories. Therefore, to assess strength of the relationship between the independent variable and the dependent variables, relative risk ratio was estimated using initiation of antenatal care within the first three months as the outcome base. The analysis was conducted using Stata v13.

### Ethical statement

This study is a secondary analysis of the 2013 NDHS, so does not require ethical approval. We were 2015 DHS Fellows, we registered and requested for access to NDHS datasets from DHS on-line archive and received approval to access and download the de-identified DHS data files. This was a pre-requisite for the Fellowship training programme and research work we conducted during the period.

## Results

The analysis was restricted to women who had a live birth in the five year period before the survey; this number turned out to be 20,467 (weighted). Their characteristics against the timing of initiation of antenatal care (ANC) is given in Table 1. On the whole, 26.8%, 61.6% and 11.6% of the women who had their last birth in the last five years initiated ANC in the first, second and third trimesters respectively. The mean gestational age at initiation of ANC is 5.3 months with a median of 4 months.

**Table 1:** Background characteristics of women by timing of antenatal care, 2013 Nigeria DHS

Characteristic	1^st^ trimester	2^nd^ trimester	3^rd^ trimester	χ^2^	*p*
**Maternal age**				**3.9**	**0.009**
Less than 18	20.1	62.2	16.9		
18-34	25.6	61.5	11.5		
35 or older	30.2	61.4	10.8		
**Maternal education**				**51.8**	**0.001**
No formal	20.9	60.3	18.9		
Primary	25.5	63.3	11.3		
Secondary and more	31.3	61.6	7.1		
**Marital status**				**1.67**	**0.156**
Married	26.7	66.9	9.5		
Formerly married	31.0	61.5	11.7		
Never married	23.5	58.2	10.9		
**Type of marriage**				**24.9**	**0.001**
Monogamy	28.2	61.5	10.4		
Polygamy	22.7	61.8	15.6		
**Parity**				**15.7**	**0.001**
One	30.1	60.3	9.6		
2-4	28.0	61.7	10.3		
5+	23.2	62.1	14.7		
**Birth order**				**12.2**	**0.001**
1^st^	30.1	60.3	9.6		
2^nd^ & 3^rd^	28.7	61.1	10.2		
4^th^ & 5^th^	25.6	63.1	11.4		
6^th^ and above	22.5	61.8	15.7		
**Pregnancy intention**				**0.82**	**0.550**
Wanted	26.8	61.3	11.9		
Mistimed	26.7	63.4	10.0		
Unwanted	25.8	64.9	9.3		
**Household Wealth**				**29.6**	**0.001**
Poor	22.7	59.4	17.9		
Middle	27.9	61.1	11.1		
Rich	28.6	63.0	8.4		
**Decision participation**				**22.6**	**0.001**
None	21.1	62.2	16.7		
One	27.4	60.9	11.8		
Two	29.4	59.7	10.9		
Three	29.6	63.8	6.6		
All four	32.6	59.6	7.8		
**Media exposure**				**28.8**	**0.001**
Not exposed	24.0	57.8	18.2		
Exposed to one	23.6	62.7	13.7		
Exposed to two	27.0	63.7	9.3		
Exposed to three	33.5	60.5	6.0		
**Zone**				**41.1**	**0.001**
North Central	41.7	50.6	7.7		
North East	21.3	61.5	17.2		
North West	13.9	68.2	18.0		
South East	31.0	61.4	7.6		
South South	26.4	63.4	10.2		
South West	32.3	61.3	6.4		
**Residence**				**11.5**	**0.001**
Urban	26.0	64.2	9.8		
Rural	27.5	59.1	13.4		
**Insurance**				**3.98**	**0.0036**
No	26.5	61.7	11.8		
Yes	37.2	57.4	5.4		
Total	26.8	61.6	11.6		

Against the background of sociodemographic factors examined here, only marital status and pregnancy intention are not significantly different with respect to initiation of ANC within the three periods of pregnancy. There appears to be a clear pattern of timing of ANC (in the first trimester) with respect to maternal level of education, household wealth level, level of participation in decision-making and level of media exposure. That is, the higher the level of maternal education the more the tendency to initiate ANC in the first trimester. Similarly, more women in the rich household (29%) initiated ANC in the first trimester compared to women in the poor household level (23%). There is also clear incremental pattern in initiation of ANC based level of involvement in household decision making. Finally, more women who were exposed to all three media channels initiated ANC in the first trimester (33%) compared to those who were never exposed (21%).

**Figure 1:**
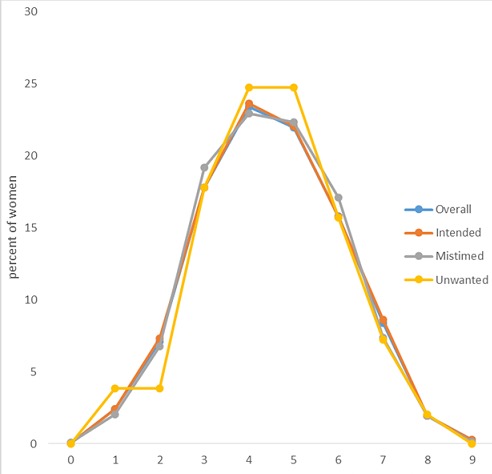
Frequency distribution of timing of ANC initiation by pregnancy intentions, 2013 Nigeria DHS

Figure 1 shows timing of ANC by pregnancy intention. Overall, it shows a close relationship of timing of ANC by pregnancy with all the curves being approximated to each other excepting in the case of unwanted pregnancy where there is a plateau between the first and the second month. The curve for the unwanted pregnancy indicates that larger percentage of women initiated ANC in the fourth and the fifth months more than those women in the other categories of pregnancy intentions.

Table 2 shows the results of multinomial logistic regression by some background sociodemographic factors. In both the two model, the findings reveal that maternal education, level of media exposure, region and place of residence are the uniform predictors of initiation of ANC; having health insurance is a significant predictor of third trimester ANC initiation relative to first to first trimester (RRR=0.72; 95%CI: 0.31-0.95). For instance, initiating ANC in the second trimester relative to the first trimester was more likely for those with primary education (RRR=1.20; 95%CI: 1.06-1.34) compared to those with secondary or more. Women who have been exposed to at least two media channels are around 30% (RRR=1.30; 95%CI: 1.15-1.46) more likely to initiate ANC in the second trimester than in the first trimester compared to those who had been exposed to all the three channels. Rural women are less likely to initiate ANC in the second trimester compared to their urban counterparts (RRR=0.8; 95%CI: 0.73-0.89).

**Table 2:** Multivariate polytomous logistic regression of factors associated with ANC initiation in the 2^nd^ and 3^rd^ trimesters relative to 1^st^ trimesters, 2013 Nigeria DHS

Characteristic	ANC initiation in the 2^nd^ trimester relative to 1^st^ trimester (Model I)		ANC initiation in the 3^rd^ trimester relative to 1^st^ trimester (Model II)	
	RRR	95% CI	RRR	95% CI
**Maternal age**				
18-34	1.00		1.00	
Less than 18	1.13	0.88-1.44	1.38	0.98-1.94
35 or older	1.01	0.89-1.14	0.85	0.71-1.03
**Maternal education**				
Secondary and more	1.00		1.00	
No formal	1.22	1.06-1.40^++^	1.68	1.36-2.08^++^
Primary	1.20	1.06-1.35^++^	1.33	1.09-1.61^++^
**Pregnancy intention**				
Unwanted	1.00		1.00	
Wanted then	0.89	0.69-1.15	0.92	0.61-1.39
Wanted later/mistimed	0.93	0.70-1.24	0.96	0.60-1.54
**Birth order**				
2^nd^ & 3^rd^	1.00		1.00	
1^st^	0.89	0.79-1.00$	0.83	0.68-1.02
4^th^ & 5^th^	1.08	0.96-1.20	1.14	0.96-1.35
6^th^ and above	1.11	0.97-1.28	1.38	1.13-1.68^+++^
**Household Wealth**				
Rich	1.00		1.00	
Poor	0.81	0.70-0.93^++^	1.02	0.82-1.27
Middle	0.92	0.81-1.04	0.92	0.76-1.12
**Decision participation**				
All four	1.00		1.00	
None	1.19	1.06-1.34	1.65	1.35-2.00^+++^
At least one	1.10	0.95-1.27	1.32	1.04-1.66^++^
At least two	1.04	0.89-1.21	1.22	0.94-1.58
At least three	1.08	0.95-1.22	0.99	0.79-1.26
**Media exposure**				
Exposed to three	1.00		1.00	
Not exposed to any	1.18	1.01-1.39^+^	2.00	1.53-2.62^+++^
Exposed to at least one	1.16	1.00-1.35^+^	1.63	1.26-2.12^+++^
Exposed to at least two	1.30	1.15-1.46^+++^	1.51	1.20-1.90^+++^
**Type of marriage**				
Monogamy	1.00		1.00	
Polygamy	0.98	0.88-1.09	1.09	0.94-1.26
**Zone**				
North Central	1.00		1.00	
North East	1.98	1.72-2.28^+++^	2.49	1.99-3.11^+++^
North West	3.59	3.08-4.19^+++^	5.04	4.00-6.35^+++^
South East	1.57	1.35-1.83^+++^	1.72	1.30-2.28^+++^
South South	2.06	1.77-2.39^+++^	3.13	2.42-4.06^+++^
South West	1.58	1.38-1.81^+++^	1.75	1.35-2.25^+++^
**Residence**				
Urban	1.00			1.00
Rural	0.80	0.73-0.89^+++^	0.72	0.61-0.85^+++^
**Insurance cover**				
No	1.00		1.00	
Yes	0.91	0.71-1.15	0.55	0.31-0.95^++^

p-values

+ <0.05; p-values

++ <0.01; p-values

+++ <0.001; p-values

$ ^+^ marginally significant at 0.05.

Other factors are not consistent predictors of second or third trimester initiation of ANC relative to first trimester within their categories. Within the categories of household wealth, levels of participation in household decision-making and region some categories are significant predictors while others are not. Regarding level of participation in household decision-making, women who did not participate at all in decision-making process are more likely to initiate ANC in the second trimester relative to first trimester (RRR=1.19; 95%CI: 1.06-1.34). Within the categories of level of participation in household decision-making and birth order are not uniform predictors; while all other factors are not significant predictors.

## Discussion

In this study we examined several factors thought to influence utilization of ANC within the first trimester of pregnancy. From literature review, several factors have been reported to be responsible for delay in initiating ANC within the first trimester of pregnancy. These include age at delivery, family income, media exposure, attitude towards pregnancy, knowledge about the danger signs of pregnancy, husband’s approval of ANC, and distance to health facility were associated with ANC service utilization at any point during pregnancy [30]. In a few African countries, health insurance coverage have been reported to influence use of ANC; Manzi reported that having enrolled into a public health insurance is associated with early ANC initiation [8]. Similarly, Arthur reported that having insurance increases odd of ANC among Ghanaian women [31]. Other researchers have identified reproductive factors and behaviour towards and during pregnancy to influence uptake of antenatal care. These factors include birth order, the desirability of a pregnancy, the ideal family size, and use of family planning [32, 33]. In this study using multinomial logistic regression, we found a range of factors influencing uptake of antenatal care within the first three months of pregnancy even though their strength, directions and uniformity varies across the three trimesters of pregnancy. Three factors emerged as the strongest, uniform and consistent predictors of ANC initiation across the two models: maternal education, media exposure, geopolitical zone and place of residence.

Maternal education has long been identified as a strong determinant of utilization of maternal health service [19, 33–46] and we are able to demonstrate its effects in predicting initiation of ANC in the first trimester of pregnancy. It is expected that educated women are more knowledgeable in health information and fully understood the benefits of utilization of ANC early in pregnancy because they are more likely to have access to health facilities and to access health information in various forms and from various channels. Additionally, issues related to access, affordability and costs of services would not serve as barriers to utilization of such services since they are more likely to be residing in urban areas where there are a range of facilities providing range of maternal health services from which to choose; furthermore, such women can afford the service since they are also more likely to be gainfully employed and earn an income. Maternal education is related to level of participation in decision-making process in the home; and because of their educational status, they can participate fully in decision making about her own health and that of other family members. Level of participation in household decision-making process is a measure of women autonomy which has also been documented to influence use maternal health services including reproductive health services Bloom [47]. Our results indicates that the influence of level of participation in household decision-making process is non-significant in the second trimester inconsistently significant within the four levels of participation in the third trimester. Women who do not in any or participated in only one decision-making process are more likely to initiate ANC in the third trimester than in the first trimester compared to women who did participate in all the four decision-making processes. The second important finding is the influence of media exposure on timing of first ANC which operates uniformly across the two models showing incremental effect with the quantum of exposure. A review of factors associated with ANC utilization indicated that exposure to mass media (especially radio and television) is strongly predictive of utilization of ANC. Women who are exposed to all the three channels of media showed incremental likelihood of first trimester initiation of ANC compared to those with lower quantum of exposure. Studies reported by Pallikadavath [48] and Sharma [49] found that watching television every week substantially increased the chances of women seeking ANC. Our results, also corroborates earlier findings of Navaneetham and Dharmalingam in India [34].

The third important finding across the two models presented in this study is the role of place of residence (urban/rural) and zone as they influence timing of ANC and is consistent with report of previous studies [32, 34, 38–46, 50]. Generally, women in rural areas use maternal health services less compared to their urban counterparts simply as a result of lack of availability, affordability and accessibility. In urban areas, there are available facilities offering wide range of services with range of costs among which women can choose. Under normal situation, women in rural areas utilize maternal health service including ANC less compared to their urban counterparts. However, with intervention programme to increase utilization rates of maternal health services there may be a reverse situation as in the case of some parts of India (in Tamil Nadu and Karnataka) [34] and Ethiopia [40]. In the Nigerian context, we believe that this rural/urban differential in the timing of first ANC could be related to the skewed distribution of health facilities as well as personnel between the urban and rural areas. It has well been documented that there is mal-distribution of health facilities and personnel in Nigeria favoring the urban areas in terms of numbers, levels of equipment and skilled personnel overseeing these facilities [51]. Furthermore, this urban-rural differential in ANC utilization could be as a result of exposure to health information and awareness on the benefits and advantages of early ANC initiation in favour of urban women [50].

As cited above, while several other factors have been found to be associated with initiation of ANC in the first trimester our results does not reveal as much as those factors as significant determinants of timing of antenatal care. These non-uniform factors in this study include pregnancy intention/desire, household wealth level and level of participation in household decision-making process. One such factor requiring attention is the status of pregnancy regarding its wantedness or desirability or intention. Though the direction and strength of relative risk ratio indicates that women who had mistimed or unwanted pregnancies have lower relative risk ratio (RRR) of initiating ANC in the second and third trimester compared to those who wanted their pregnancies, our findings does not support this claim since the RRR are not statistically significant. Our result supports the findings of Gage et al [52] who documented the lack of significant role of pregnancy wantedness in determining the timing of first ANC visit in Kenya and Namibia. Pregnancy desire/intention has been shown to have mixed influence of the timing of ANC and maternal behaviours. The study by Exavery et al [53] showed that RRR of second and third trimester initiation of ANC are larger for mistimed and unwanted pregnancies than for wanted or planned pregnancies. However, in a five-country study, Marston and Cleland [54] reported that only in one country was pregnancy desire not strongly associated with lack of ANC in the first six months of pregnancy. Furthermore, Kost et al [33] have previously reported that pregnancy intention and sociodemographic factors are strong predictors of maternal behaviors including early initiation of ANC, recognize danger signs in pregnancy. Similar mixed findings of the influence of pregnancy intention and timing of first ANC was reported by Eggleston in Ecuador where the author concluded that there was no association between pregnancy intention and use of ANC and initiation of ANC in the first trimester of pregnancy in the case of mistimed pregnancy but not unwanted pregnancy [55].

Unexpectedly, with regard to wealth level, it is found that, women in rich households are more likely to initiate ANC in the second trimester than in the first compared to women in poor households and that this relationship disappears with regard to third trimester. This is unexpected among women from rich households since lack of money for transport and other hospital consumables are frequently cited by women as factors in delaying utilization of maternal health service including timely initiation of ANC but we believe that financial constraints should not deter early ANC initiation among women from rich households [43, 56–58].

### Strength and limitations

An important strength of this study is that it used a large nationally-representative sample size and to the best of our knowledge this study represents the first of its kind in Nigeria using a nation-wide data. Despite this, we believe there are limitations to this study. Firstly, the survey did not collect information why women initiated ANC late. Various reasons could be responsible for delay in starting ANC late that ranges from socioeconomic such as cost of service, cost of transport and also opportunity cost of using ANC; sociodemographic such as place of residences (rural versus urban) and the health services factors such quality, perception and satisfaction of services received especially for those who had experience with the health service. Again, this type of study can only generate associations/correlations between delay in ANC utilization and some factors; it is limited in its design to establish causality between the outcome variable and the explanatory variables.

## Conclusion

Several factors were found to delay initiation of ANC and in sum the uniform and consistent predictors for delayed ANC visit are basically sociodemographic; maternal education, media exposure, place of residence and zone of residence. Maternal education remains a powerful predictor of utilization of maternal health services.

### What is known about this topic

- Timing of ANC has a significant influence on outcome of the pregnancy;
- Delayed initiation in ANC is associated with poor pregnancy outcomes;
- Some factors have been reported as determinants of timing of ANC in Nigeria using hospital-based studies.

### What this study adds

- Only 27% and 62% of pregnant women initiated ANC in the first and second trimester respectively;
- In the Nigeria context, maternal education, media exposure, zone of residence and place of residence are uniform predictors of initiation of ANC in the first trimester.
